# Assessing the Relationship Between a Composite Score of Urban Park Quality and Health

**DOI:** 10.5888/pcd15.180033

**Published:** 2018-11-08

**Authors:** Lauren E. Mullenbach, Andrew J. Mowen, Birgitta L. Baker

**Affiliations:** 1 Department of Recreation, Park and Tourism Management, The Pennsylvania State University

## Abstract

**Introduction:**

Walkable access to parks, sufficient park acreage, and investments in park and recreation resources are 3 indicators of quality city park systems. Few studies, however, have examined the collective effects of these indicators on public health outcomes.

**Methods:**

Combining 3 nationwide public data sets, this study modeled the relationships between a composite score of urban park system quality effects on physical activity and self-reported health while controlling for demographic and lifestyle variables. Data were obtained from the Centers for Disease Control and Prevention’s 500 Cities Project, the Trust for Public Land’s City Park Facts Report, and the US Census Bureau.

**Results:**

Regression analyses indicated that the composite park quality score was significantly related to both physical activity levels and physical health across a sample of 59 cities. Higher scores were associated with fewer physically inactive residents but were not significantly associated with better physical health.

**Conclusion:**

Assessing the collective contribution of park access, park acreage, and investment suggests that improvements to a city’s composite score may correspond with greater physical activity, but more research is needed to establish the long-term relationships between park system quality and physical health.

## Introduction

Use of public parks is associated with many health benefits, including increased physical activity levels, reduced stress, and better self-reported health (1–3). Prior research has established that walkable park access, park availability, and adequate park funding are particularly important contributors to health outcomes (4–6), but these have yet to be tested together empirically. For example, living within walkable access to parks is associated with significantly higher physical activity and park use (7) and better mental health (8). One study also found that psychological sense of community increased as residential distance from the park decreased (9). Therefore, having a park within walking distance can offer multiple health benefits. The amount of available park land and a city’s physical park assets are also associated with better physical and mental health outcomes (10). A prior study found that more park acres per capita and higher park density were associated with lower levels of obesity and higher levels of exercise and physical activity (11). Finally, adequate funding is required to provide both this walkable proximity to parks and sufficient acreage, in addition to supporting the in-park amenities, programming, and maintenance that draw users. Financial expenditures on parks and recreation can lead to increases in physical activity and sports participation (12–13).

Because of potential linkages to health, we used the Trust for Public Land’s composite measure of access, acreage, and investment, known as ParkScore (14), to test its relationship with physical activity and self-reported health across a sample of US cities. An earlier study (11) examined the individual impact of these indicators on urban health, but their collective impact on health could be even greater. We assessed ParkScore’s relationship to city-level physical activity and health while controlling for a range of city-wide demographic and lifestyle characteristics.

## Methods

Using the 2014 City Park Facts Report (15) from the Trust for Public Land (16), the public health database from the Centers for Disease Control and Prevention (CDC) 500 Cities Project (17), and data from the US Census Bureau, we examined the relationships among walkable park access, spending on parks and recreation, park assets, and public health for 59 cities in the United States.

### Data sources

**Health outcomes and covariates.** The 500 Cities Project (17) from CDC is a nationwide public health project that has produced several free, open-access databases. The data are from the Behavioral Risk Factor Surveillance System (BRFSS), an annual nationwide health survey, and the results have been grouped by city. BRFSS data are typically grouped by metropolitan statistical area (MSA); this project, however, partnered with the Robert Wood Johnson Foundation to aggregate data to the city-boundary level for the 500 largest cities in the United States. Using small area estimation, the individual-level data were aggregated to the city-level. The city-level database includes 27 measures of chronic disease, including obesity, physical health, and physical activity, which are modeled predictions based on samples of BRFSS respondents and weighted based on each city’s demographic profile. Additional information on this project can be found online (17). Data for the 500 Cities Project were collected during the 2014 BRFSS. The two outcome variables from BRFSS chosen for this study were the number of days in the last month that residents felt physically unwell, as a measure of physical health (aggregated to proportion reporting >14 days) and proportion of the population getting no leisure-time physical activity. Health variables that served as controls because of their demonstrated relationships with self-reported health were prevalence of smoking and obesity in each city (18,19).

**Walkable park access, park acreage, and park and recreation spending.** The Trust for Public Land (TPL) releases an annual report on the economic conditions of city park and recreation systems across the country. The City Park Facts Reports are free and available to the public on the TPL website (16). More about how TPL determines city boundaries is also available on the website. We used the 2014 City Park Facts Report (15) to maintain consistency in year with the BRFSS data set. These yearly TPL reports contain many variables describing the assets and spending patterns of the country’s 100 largest cities. The predictor variable in this study was ParkScore, a composite measure of park access, park spending, and park acreage created by TPL. TPL calculates a score for each of the 100 largest cities based on specific criteria; scores range from 0–100, with 100 being a perfect score. The ParkScore is the sum of 3 equally weighted scores in 1) access, 2) acreage, and 3) investments and amenities. Access is based on the percentage of the city population living within a 10-minute walk to a park. Acreage includes measures of median park size and parkland as a percentage of city land area. Investment and amenities include measures of spending on parks and recreation per resident and a per capita average of amenities such as basketball hoops, dog parks, playgrounds, and recreation centers. More information on how TPL calculates scores is available elsewhere (20). This composite score enabled us to test the effects of our 3 predictor variables at once, which was important for this sample.

Certain demographic characteristics of each city in the sample were obtained from the Census Bureau’s American Community Survey (ACS) for 2014 (21). Race was represented as percentage of the adult population that is black or African American and ethnicity was represented by percentage of the adult population that is Hispanic or Latino. Although not a comprehensive measure of race and ethnicity, these 2 races/ethnicities are the largest minority groups in the United States, are often studied in regard to physical activity and park use (22,23), and experience significant health disparities compared with the white non-Hispanic population (24,25). Median income for the city was obtained from Data USA (26). Finally, education was represented as the percentage of the adult population with a college degree, according to the 2014 ACS. City-wide median income and education level were converted to Z scores and averaged together to create a socioeconomic status (SES) variable. Prevalence of smoking and obesity were expressed as percentage of the adult population in the city who smoke and meet criteria for obesity, respectively.

### Analysis

First, descriptive statistics were calculated for sociodemographic characteristics, city population, smoking and obesity prevalence, ParkScore, physical health, and leisure-time physical inactivity (Table 1). Pearson correlations were run to examine the strength of the relationships between outcome and predictor variables. ParkScore as a predictor of physical inactivity and perceived health was then tested using 2 weighted least squares regression models, controlling for city-wide SES, race, ethnicity, smoking rates, and obesity levels. The health outcome variables are already adjusted for age, eliminating the need to control for age with an additional variable; information on age-adjustment procedures is also available (14). Analytic weights were applied to both models to account for variation in the precision of estimates (eg, larger cities construct estimates from larger samples than do smaller cities). Weights were calculated by using the inverse of the standard error of the confidence intervals for estimates of physical inactivity and physical health. All analyses were conducted by using the IBM Statistical Package for the Social Sciences version 24.

**Table 1:** Descriptive Statistics, 59 US Cities, 2014

Characteristic	Mean (SD)	Median	Range
Proportion Hispanic^a^^,^ b	26.6 (19.8)	23.3	3.7–79.7
Proportion Black/African American^a^	21.3 (18.3)	16.0	1.2–80.9
Education: bachelor’s degree or higher	31.5 (9.8)	29.7	11.8–57.9
Median income, $	53,136 (14,584)	50,721	25,980–105,355
Obesity prevalence^c^	29.9 (5.5)	30.4	15.6–45.2
2010 population	607,256 (929,885)	373,903	204,214–8,175,133
ParkScore^d^	52.2 (13.6)	51.0	26.0–82.0
Physical inactivity^e^	25.0 (5.1)	25.7	13.6–37.6
Physical health^f^	12.8 (2.4)	13.0	7.9–18.4

a Percentage of population from 2014 American Community Survey (21) estimates.

b Median used for Hispanic population because of its negatively skewed distribution, to avoid extreme values influencing the mean.

c Age-adjusted prevalence of obesity in adult population.

d Scores range from 0 to 100.

e Operationalized as modeled prediction of proportion of the population getting no leisure-time physical activity.

f Operationalized as modeled prediction of proportion of the population who reported >14 days physically unwell in the last month.

## Results

### Sample characteristics

Of the 500 cities from the CDC data set and 100 cities from the TPL data set, 98 overlapped, and of those, 59 had ParkScores, providing a sample of 59 cities. Those 59 cities represent 31 states and the District of Columbia. Mean ParkScore was 52 (standard deviation [SD] = 13.6). The mean physical inactivity score, representing percentage of the population that gets *no* leisure-time physical activity, was 25% (SD = 5.1%). And the mean physical health score, representing modeled predictions of the proportion of the population who reported feeling physically unwell >14 days over the last month, was 12.8% (SD = 2.4%). Table 1 shows full descriptive statistics.

Correlations were strong between predictor and outcome variables. ParkScore was significantly related to both physical inactivity (r = −.55, *P* < .001) and physical health (r = −.49, *P* < .001). The negative correlation indicated that higher ParkScores are associated with smaller proportions of the population having no physical activity and a smaller proportion of the population reporting they felt physically unwell >14 days in the last month. Regression models assessed the associations between ParkScore and physical inactivity and physical health. Smoking prevalence was removed from both models, because of problems with multicollinearity (variance inflation factor = 8.34 and 7.79 for physical inactivity and physical health, respectively). When controlling for SES, race, ethnicity, city population, and obesity prevalence, ParkScore significantly predicted physical inactivity (β = −.06, *t* = −2.186, *P* = .033, R^2^ = .76) but did not significantly predict physical health (β = −.018, *t* = −1.147, *P* = .257, R^2^ = .71) (Tables 2 and 3).

**Table 2:** Multiple Regression of ParkScore Predicting Physical Inactivity^a^^,^ b, 59 US Cities, 2014

Independent Variable	β (SE)	Standardized β	*P* value
Proportion Hispanic^c^	.08 (.02)	.32	.001
Proportion Black/African American^c^	.08 (.03)	.32	.01
Socioeconomic status^d^	−.95 (.68)	−.18	.17
Obesity prevalence^c^	.34 (.12)	.40	.01
2010 population^e^	.00000062 (.00000026)	.16	.02
ParkScore^f^	−.06 (.03)	−.19	.03

a Model summary: R^2^ = .76, F(6, 51) = 31.62, *P* < .001.

b Operationalized as modeled prediction of the proportion of the population getting no leisure-time physical activity.

c Values range from 0 to 100, representing percentage of the city population.

d Average of Z scores for median income and percentage of the city population with a college degree.

e Population from 2010 Census.

f Scores range from 0 to 100.

**Table 3:** Multiple Regression of ParkScore Predicting Physical Health^a–c^, 59 US Cities, 2014

Independent Variable	β (SE)	Standardized β	*P* value
Proportion Hispanic^d^	.04 (.01)	.29	.01
Proportion Black/African American^d^	.06 (.02)	.49	<.001
Socioeconomic status^e^	−1.55 (.36)	−.60	<.001
Obesity prevalence^d^	−.03 (.05)	−.08	.60
2010 population^f^	.000000147 (.00000015)	.07	.33
ParkScore^c^	−.02 (.02)	−.11	.26

a Model summary: R^2^ = .71, F(6, 51) = 23.76, *P* < .001.

b Operationalized as modeled prediction of the citywide proportion of the population who reported being physically unwell >14 days in the last month.

c Scores range from 0 to 100.

d Values range from 0 to 100, representing percentage of the city population.

e Average of Z scores for median income and percentage of the city population with a college degree.

f Population from 2010 Census.

## Discussion

Our results illustrate the potential contribution of a quality city park system to physical activity. We found that in cities with robust park systems (as determined by their ParkScores), residents were more engaged in physical activity. For example, residents from cities with higher ParkScores were less likely to be physically inactive, even while controlling for other lifestyle factors, such as SES, race, ethnicity, and obesity. These results are consistent with prior research that looked at park acreage and its impact on obesity and physical activity (11), and our study shows the additional impact of 2 other domains of park capacity, park access and investment, as part of the ParkScore (although the individual contributions of these factors were not assessed in this study).

These results have implications for city governments, park agencies, and park nonprofit organizations. According to our model, if a city increases its ParkScore by 10 points (out of a possible 100 points) while holding all else constant, the percentage of the population getting no leisure-time physical activity could decrease by 0.64%. At a population level, this effect could be quite noticeable. For example, if Atlanta — a city with a 2010 population of 420,003 — increased its 2014 ParkScore of 44 points to 54 points, 2,688 additional people could engage in leisure-time physical activity. Although this study was cross-sectional and therefore did not look at increases directly, it is possible that enhancements made to proximity, acreage, and funding could provide physical activity benefits across these cities.

### Limitations

We acknowledge several study limitations. First, our results represent a snapshot in time; all data are from 2014, and therefore causality cannot be determined. Additionally, correlations indicate that 25% of physical health is associated with ParkScore, leaving about 75% of physical health associated with other factors not measured in this study, such as genetics, lifestyle, occupation, or diet. The way physical health was measured in this study may limit its usefulness. The criteria for determining physical health are restrictive, and the range in values within the sample was small compared with other variables in the models. A limited measure of proportion of population feeling unwell for >14 days in the last month may not be the best indicator of physical health in a city. Additionally, physical health may take longer to achieve and be more resistant to change than physical activity. A longitudinal study may be better able to capture the possible effects of park system quality and physical activity on physical health. Finally, a sample size of 59 cities is relatively small.

Despite these limitations, our findings have implications for future research that integrates park capacity data with health data. More effort could be devoted to connecting secondary parks, recreation, and health data, especially from this type of paired data set (27). Given that city-level health data are now available, fulfilling prior promises to connect physical activity and health at more precise levels (27), their use could be expanded. In addition to CDC’s 500 Cities Project, more detailed measures of physical activity and health could be incorporated into park assessments and vice versa: park use and leisure-time physical activity items could be incorporated into public health measures. For researchers, the development of the CDC city-level data set is significant, because of its potential to be matched with city-level park excellence data, allowing for a more direct comparison between park metrics and health outcomes.

Future work in this area is encouraged and could become part of a wider research agenda. For instance, in addition to physically active use of parks, social indicators related to park use should not be forgotten in this research agenda. Nor should other chronic disease or public health outcomes be neglected. Additionally, more frequent tracking of public health related to park use at the city level is needed beyond cross-sectional data. For instance, longitudinal studies tracking ParkScore and health outcomes over time would be an interesting, and potentially compelling, examination of the impact of city park systems on chronic disease. Tracking of residents’ health and physical activity over time could be paired with changes in their environment, access to parks and recreation resources, and changes in park investment to examine relationships over time. 

## Conclusion

Given the growth in city populations in recent decades, and projected increases in the future, the relative health of cities’ built and natural environments can affect a large portion of the country’s population. As such, the contribution of urban parks to sustaining and improving public health is important to demonstrate to park agencies, city officials, and lawmakers. Future research linking park access, acreage, and investment with the prevalence of chronic disease is needed to confirm the importance of each of these indicators (as well as other, more salutogenic, indicators) in relation to other health benefits for urban residents. As cities work to promote health for *all* of their residents, the health contribution of their park systems should not be overlooked.
